# Discordance of PD-L1 Expression at the Protein and RNA Levels in Early Breast Cancer

**DOI:** 10.3390/cancers13184655

**Published:** 2021-09-16

**Authors:** Ioannis Zerdes, Vaia Karafousia, Artur Mezheyeuski, Maria Stogiannitsi, Raoul Kuiper, Pablo Moreno Ruiz, George Rassidakis, Jonas Bergh, Thomas Hatschek, Theodoros Foukakis, Alexios Matikas

**Affiliations:** 1 Department of Oncology-Pathology, Karolinska Institutet, 171 77 Stockholm, Sweden; ioannis.zerdes@ki.se (I.Z.); vkarafousia@gmail.com (V.K.); stogiam@hotmail.com (M.S.); pablo.moreno.ruiz@ki.se (P.M.R.); georgios.rassidakis@ki.se (G.R.); jonas.bergh@ki.se (J.B.); thomas.hatschek@ki.se (T.H.); theodoros.foukakis@ki.se (T.F.); 2 Breast Center, Theme Cancer, Karolinska University Hospital, 171 77 Stockholm, Sweden; 3 Department of Immunology, Genetics and Pathology, Uppsala University, P.O. Box 516, 751 20 Uppsala, Sweden; artur.mezheyeuski@igp.uu.se; 4 Department of Laboratory Medicine, Karolinska Institutet, 171 77 Stockholm, Sweden; raoul.kuiper@ki.se; 5 Department of Pathology and Cytology, Karolinska University Hospital, 171 77 Stockholm, Sweden

**Keywords:** breast cancer, heterogeneity, in situ hybridization, multiplex immunohistochemistry, PD-L1

## Abstract

**Simple Summary:**

Despite the increasing use of checkpoint inhibitors for early and metastatic breast cancer, Programmed Death Ligand 1 (PD-L1) remains the only validated albeit imperfect predictive biomarker. Significant discordance in PD-L1 protein expression depending on the antibody used has been demonstrated, while the weak correlation and discordant prognostic information between protein and gene expression underscore its biologic heterogeneity. In this study, we use material from two patient cohorts of early breast cancer and multiple methodologies (immunohistochemistry, RNA fluorescent in situ hybridization, immunofluorescence, bulk gene expression, and multiplex fluorescent immunohistochemistry) to demonstrate the significant discordance in PD-L1 expression among various methods and between different areas of the same tumor, which hints toward the presence of spatial, intratumoral and biological heterogeneity.

**Abstract:**

We aimed to assess if the discrepant prognostic information between Programmed Death Ligand 1 (PD-L1) protein versus mRNA expression in early breast cancer (BC) could be attributed to heterogeneity in its expression. PD-L1 protein and mRNA expression in BC tissue microarrays from two clinical patient cohorts were evaluated (105 patients; cohort 1: untreated; cohort 2: neoadjuvant chemotherapy-treated). Immunohistochemistry (IHC) with SP142, SP263 was performed. PD-L1 mRNA was evaluated using bulk gene expression and RNA-FISH RNAscope^®^, the latter scored in a semi-quantitative manner and combined with immunofluorescence (IF) staining for the simultaneous detection of PD-L1 protein expression. PD-L1 expression was assessed in cores as a whole and in two regions of interest (ROI) from the same core. The cell origin of PD-L1 expression was evaluated using multiplex fluorescent IHC. IHC PD-L1 expression between SP142 and SP263 was concordant in 86.7% of cores (*p* < 0.001). PD-L1 IF/IHC was weakly correlated with spatial mRNA expression (concordance 54.6–71.2%). PD-L1 was mostly expressed by lymphocytes intra-tumorally, while its stromal expression was mostly observed in macrophages. Our results demonstrate only moderate concordance between the various methods of assessing PD-L1 expression at the protein and mRNA levels, which may be attributed to both analytical performance and spatial heterogeneity.

## 1. Introduction

Pivotal randomized studies have demonstrated the efficacy of combined chemoimmunotherapy using immune checkpoint inhibition for both early and metastatic triple-negative breast cancer (TNBC) [1,2,3,4]. Robust predictive biomarkers are currently lacking, with PD-L1 protein expression showing inconsistent clinical utility for benefit at the metastatic setting [1,5] and varying concordance when assessed by different clones [6]. Other explored biomarkers such as an abundance of tumor-infiltrating lymphocytes or immune gene expression have not shown clinical utility [7,8]. In addition, neoadjuvant chemoimmunotherapy improves pathologic complete response rates regardless of PD-L1 status compared with chemotherapy alone [2,3]. These observations raise questions about whether PD-L1 protein expression is adequate for guiding the use of costly and potentially toxic immune checkpoint inhibitors.

Potential causes for the reported inconsistency include assay performance [6]; a potentially lesser role of immunoediting at the primary tumor compared with the distant metastasis, further supported by the lower PD-L1 positivity rates at metastasis compared to primary tumor [9] and the fact that PD-L1 positivity at the metastasis carried potentially stronger predictive information in the IMPassion130 trial [7]; and heterogeneity of PD-L1 expression. In support of the latter, we have previously demonstrated that PD-1/PD-L1 expression is associated with discrepant prognostic values depending on the level of expression, mRNA, or protein [10,11]. This fact hints toward heterogeneity (spatial, biological, and/or technical) of PD-L1 expression, a non-fully addressed topic in BC.

The aim of this study was to investigate the heterogeneity of PD-L1 expression at the mRNA and protein level in early BC. We, therefore, applied multi-level testing of PD-L1 expression in two early BC patient cohorts (both treated and untreated), including: (a) immunohistochemical (IHC) staining with two different commercially available antibody clones; (b) RNA fluorescent in situ hybridization (RNA-FISH) combined with immunofluorescence (IF) for simultaneous detection of mRNA-protein expression on the same tissue section; (c) bulk PD-L1 gene expression; and (d) multiplex fluorescent immunohistochemistry.

## 2. Materials and Methods

### 2.1. Patient Cohorts

Cohort 1: This retrospective patient cohort consists of patients diagnosed with primary BC in Stockholm between 1997 and 2005 who were selected using the regional cancer registry. The cohort has been previously described in detail [12]. Τhe ethics committee at Karolinska Institutet, Stockholm, Sweden (Dnr 2006/394-31/3, 2006/1183-31/2 and amendments 2016/1505-32, 2018/789-32, 2018/790-32) has approved the analyses for this cohort and decided that no additional patient informed consent was needed.

Cohort 2: The PROMIX trial is an academic, non-randomized, single-arm, multicenter phase II study that was conducted in 5 centers in Sweden (Clinicaltrials.gov identifier NCT00957125). Patients with locally advanced, HER2-negative BC were enrolled and treated with neoadjuvant epirubicin 75 mg/m^2^ in combination with docetaxel 75 mg/m^2^, both administered every three weeks for six cycles. Bevacizumab 15 mg/kg every three weeks was added to cycles 3–6 in case of non-complete clinical response. Biopsies were taken at baseline, after cycle 2, and at surgery. Correlative studies on gene expression have been previously published [13,14]. The trial, including the correlative analyses, was approved by the Ethics Committee at Karolinska Institutet (2007/1529-31/2), which had jurisdiction for all participating centers and by the Swedish Medical Product Agency. All patients provided written informed consent prior to enrollment.

### 2.2. Tissue Microarrays (TMA), IHC Methods and Scoring of Commercially Available Antibodies

TMA were generated from primary tumors of all patients in both cohorts, using an automated tissue microarrayer (VTA-100, Veridiam, Oceanside, CA, USA). Representative tumor-rich areas, previously annotated by a certified pathologist, were selected from the formalin-fixed paraffin-embedded (FFPE) patient tissue blocks. Each TMA consisted of cores with a diameter of 1 mm and included duplicate cores per tumor/patient. Tissue sections (thickness: 4 μm) were prepared from the TMA in cohort 1 for staining with primary monoclonal SP142 and SP263 PD-L1 antibody clones (Ventana, Roche, Basel, Switzerland) using Ventana automated staining system (Roche, Basel, Switzerland) according to the manufacturer’s protocol. Reactive lymphoid tissue of the tonsil was used as a control. PD-L1 was evaluated in both tumor and immune cells as previously described [12]. A core was considered as PD-L1-positive (PD-L1+) when at least one cell with membranous immunostaining was detected in at least one TMA core. TMA cores with poor tissue quality were excluded from the analysis.

### 2.3. Cell Lines and FFPE Cell Blocks

The BC cell lines MCF7 (estrogen receptor-positive (ER+)/luminal), MDA-MB-231, and BT549 (TNBC/basal-like), with previously described PD-L1 IHC expression status and culturing conditions [15], were used for the generation of FFPE cell blocks. All cell lines have been purchased from ATCC (Manassas, VA, USA). Control slides containing FFPE cultured cell pellets of HeLa human cells were provided by ACD Bio-Techne (Minneapolis, MN, USA).

### 2.4. Immunofluorescence

Cell line FFPE block sections (thickness: 4 μm) were prepared, baked at 60 °C for 1 h, deparaffinized in xylene for 15 min, and rehydrated in graded alcohols. The pre-treatment procedure included the application of target antigen retrieval (AR9, Perkin Elmer, Waltham, MA, USA) under microwave treatment. Slides were subsequently washed, blocked, and incubated overnight with primary PD-L1 antibody (SP142, Abcam; 1:100 in TBS-Tween). Incubation with the Alexa Fluor 488 (Invitrogen, Carlsbad, CA, USA) conjugated secondary antibody followed, and slides were then counterstained with DAPI and mounted.

### 2.5. RNA In Situ Hybridization Combined with Immunofluorescence

*Staining and imaging process:* RNA in situ hybridization was performed using RNAscope^®^ Multiplex Fluorescent v2 Assay (Bio-Techne, Minneapolis, MN, USA), which was also combined with IF staining for the simultaneous detection of protein according to the manufacturer’s instructions and interaction with the manufacturer. Tissue sections (thickness: 4 μm) were prepared from the TMAs, baked at 60 °C for 1 h, deparaffinized in xylene, and rehydrated in graded alcohols. The pre-treatment procedure included the application of hydrogen peroxide, target retrieval, and protease plus reagents. RNAscope probes were then hybridized to target RNA molecules and control probes for 2 h at 40 °C. TMAs were initially stained with three different target probes, including *ESR1*, *CD274,* and *ERBB2*, but for the scope of this project, only the RNAscope Hs-CD274-C2 (ACD. cat. 600861-C2) mRNA probe’s fluorescent channel signal was selected for further analysis. The positive control probes provided by Bio-Techne included a ready-to-use mixture of the three probes (i) DNA-directed RNA polymerase II subunit RPB1 (PolR2A), coupled in C1 channel with Opal 570 fluorophore (1:1500), (ii) Cyclophilin B (PPIB), coupled in C2 channel with Opal 690 flurophore (1:1500) and (iii) ubiquitin C (UBC-C3), detected in C3 channel with Opal 520 flurophore (1:3000) all tested in HeLa human cells (Figure S1); the negative control probe was DapB (*B. subtilis* gene dihydrodipicolinate reductase). The hybridization signals were then amplified and developed via the assignment of Opal^TM^ fluorophores (Akoya Biosciences, Marlborough, MA, USA) to the respective channels. For the combined immunofluorescence antibody staining, slides were subsequently washed, blocked with 5% TBS-Tween and goat serum, and incubated overnight with primary PD-L1 antibody (SP142 Abcam; 1:100 in TBS-Tween). Incubation with HRP-conjugated secondary antibody and assignment of Opal^TM^ 620 fluorophore was then followed. Slides were counterstained with DAPI (ACD) and mounted with fluorescent mounting medium (Prolong Gold Antifade Mountant, Dako, Santa Clara, CA, USA). The workflow of the sequential PD-L1 mRNA and protein staining using RNAscope and IF is also depicted in Figure S2. Image acquisition of the stained TMAs was performed using the multispectral Vectra^®^ 3 Automated Quantitative Pathology Imaging System (Akoya Biosciences, Marlborough, MA, USA) at 20× magnification, and the images were further processed using the companion inForm^®^ software (Akoya Biosciences, Marlborough, MA, USA). Sections from the FFPE cell line blocks were imaged on a Carl Zeiss LSM 880 confocal microscope at a 63× magnification, and z-stacks of 5–7 um thickness were obtained.

*Signal quantification and scoring:* PD-L1 protein expression derived from IF staining was assessed in two different randomly selected regions of interest (ROI), containing at least 50 cells (tumor or immune cells) each. PD-L1-positive cells (presence of cell membranous staining regardless of cell of origin) were manually counted, and the respective percentage of PD-L1 positivity was then determined for each ROI. In order to further evaluate the expression by PD-L1 IF using the SP142 antibody clone, two different quantitative approaches were performed: a) 1 positive cell was considered as the threshold for PD-L1 positivity of the ROI, and b) the absolute count of cells with PD-L1+ expression within the ROIs. The mRNA signal for *CD274* in each image was selected among the different channels along with the one of DAPI for the identification of cell nuclei. Quantification of the mRNA dots was performed in the same areas as the IF in accordance with the manufacturer’s assessment instructions using the semi-quantitative ACD (advanced cell diagnostics) 5-scale score as follows: 0 = No staining or <1 dot/10 cells; 1 = 1–3 dots/cell; 2 = 4–9 dots/cell and none or very few dot clusters; 3 = 10–15 dots/cell and/or <10% dots are in clusters; 4 = >15 dots/cell and/or >10% dots are in clusters. Image analysis and expression assessment was performed using ImageJ software v, 1.48 (NIH, Bethesda, MD, USA).

### 2.6. Multiplex Fluorescent Immunohistochemistry

Tissue sections (thickness: 4 μm) were prepared from TMA FFPE blocks in cohort 1 for subsequent staining with multiplex fluorescent IHC. A modified version of the custom-based 7-color IHC kit (OpalTM 7 Solid Tumor Immunology Kit, PerkinElmer, Waltham, MA, USA) and the fully automated Leica Bond RX^m^ (Leica Biosystems, Buffalo Grove, IL, USA) Advanced Staining IHC instrument was used for the multiplex staining, as previously described [16,17]. The antibody panel consisted of 6 immune markers including CD4 (4B12 clone, 1:100 Agilent, Santa Clara, CA, USA), CD8a (C8/144B clone, 1:200 Thermo Fisher, Waltham, MA, USA), PD-L1 (SP142 clone, 1:400, Abcam, Cambridge, UK), PD-1 (NAT105 clone, 1:100, Abcam), FoxP3 (D6O8R clone, 1:300, Cell Signaling, Danvers, MA, USA), CD68 (PG-M1 clone, 1:400, Agilent) and a combination of primary antibodies against the epithelial tissue markers E-cadherin (36/E clone, 1:2000, BD Biosciences, Franklin Lakes, NJ, USA) and pan-cytokeratin (C-11 clone, 1:500, Abcam and AE1/AE3 clone, 1:400, Dako, Santa Clara, CA, USA) while 4′,6-diamidino-2-phenylindole (DAPI) was used in order to visualize the nuclei. The slides were subsequently mounted with Prolong Diamond Antifade Mountant (Thermo Fisher, Waltham, MA, USA), and image acquisition was performed using the Vectra^®^ Polaris™ Automated Quantitative Pathology Imaging System (Akoya Biosciences, Marlborough, MA, USA). Image analysis, thresholding, multispectral unmixing, and machine-learning tissue segmentation algorithm were applied using the Phenochart (Akoya Biosciences, Marlborough, MA, USA) and inForm^®^ image analysis software (Akoya Biosciences, Marlborough, MA, USA). Manual annotation of different tissue types (tumor, stroma, and blank areas), cell segmentation, and image curation to exclude artifacts, staining defects, and accumulation of immune cells in necrotic areas and intraglandular structures was performed by a certified pathologist (AM) or trained researchers (IZ, PMR). Cell density (cells/mm^2^) of every single marker per tissue compartment (tumor and stromal areas) was then computed for each sample based on predefined positivity threshold, while co-expression patterns (i.e., PD-L1 + CD4 + cells) were also calculated, as previously reported [16].

### 2.7. Gene Expression Analysis

Total RNA was extracted from fresh-frozen tumors using the Qiagen RNeasy Mini Kit (Qiagen, Hilden, Germany), and samples were hybridized using the Rosetta/Merck Human RSTA Custom Affymetrix 2.0 microarray (Gene Expression Omnibus accession number: GSE48091), as previously described [12,18]. A log-transformed expression value for each probe was obtained after background correction and normalization using the RMA method as well as the aroma.affymetrix R package [19]. When multiple probe sets (*CD274*) were mapped to the same Entrez Gene ID, the one with the highest interquartile range was selected.

### 2.8. Statistical Analysis

Summary tables (descriptive statistics and/or frequency tables) were provided for all variables, as appropriate. Continuous variables were summarized with descriptive statistics. The heterogeneity of PD-L1 expression at the protein and mRNA level was evaluated separately using different metrics related to its intratumoral expression in the two cohorts. These included PD-L1 expression: (i) between two different TMA cores of the same patient (only for cohort 1) and (ii) within two different ROIs of the same TMA core. Differences in terms of continuous variables were assessed by the Mann–Whitney U test, while correlations were assessed Spearman’s rank correlation coefficient wherever appropriate. The concordance of dichotomous variables was assessed using the Pearson chi-square test. Due to the exploratory nature of the study, no sample size calculation was performed. All statistical tests were two-sided, and *p* < 0.05 was considered statistically significant. All analyses were performed on the IBM SPSS statistical software version 25.0 (IBM, New York, NY, USA), and GraphPad Prism version 8.0 (GraphPad Software Inc., San Diego, CA, USA) was used for graphical representation.

## 3. Results

### 3.1. Patient Cohorts, Characteristics, and Tissue Availability

In cohort 1, 69 individual untreated patients were included, corresponding to a total of 134 TMA cores (65 patients with duplicate TMA cores and 4 with single tissue core). In cohort 2, tissue samples had been obtained from 36 individual neoadjuvant chemotherapy (NACT)-treated patients, corresponding to a total of 56 TMA cores. Out of these 56 cores, 39 (69.6%) had been obtained from tissue at the time of surgery after 6 cycles of NACT, 1 (1.7%) mid-treatment after 2 cycles of NACT, while information was missing for 16 (28.5%). The detailed baseline characteristics of patients in both cohorts and the workflow of the study are presented in Table S1 and Figure S3, respectively.

### 3.2. Feasibility and Analytical Validity of PD-L1 mRNA, Protein and Simultaneous Detection in Breast Cancer Cell Lines

**Figure 1:**
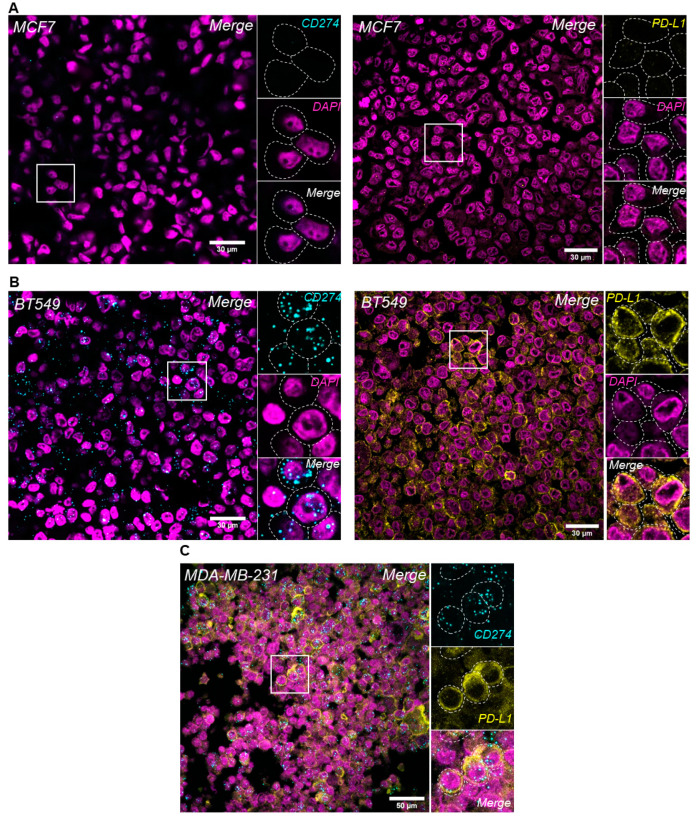
Evaluation of PD-L1 expression levels in breast cancer cell lines. PD-L1 in situ mRNA and protein expression using RNAscope^®^ and IF, respectively, in MCF7 (**A**) and BT549 (**B**) cells. Simultaneous detection of PD-L1 protein and mRNA expression using sequential RNA-FISH/IF method in MDA-MB-231 BC cell line (**C**); original magnification: 630×.

PD-L1 mRNA and protein expression were evaluated in BC cells and were concordant both in the PD-L1-negative (MCF7) and -positive (BT549) cell lines (Figure 1, upper and middle panel). The analytical validity of RNA in situ hybridization (RNA-ISH) using RNAscope^®^ combined with immunofluorescence (IF) for PD-L1 mRNA and protein levels in the same FFPE tissue section was evaluated in this study. Sequential RNA-FISH-IF was performed in the PD-L1-positive MDA-MB-231 BC cell line, and high expression levels of both PD-L1 protein and mRNA were noted in the same cells (Figure 1, lower panel).

### 3.3. PD-L1 Protein Expression with SP142 and SP263 IHC Antibodies

**Figure 2:**
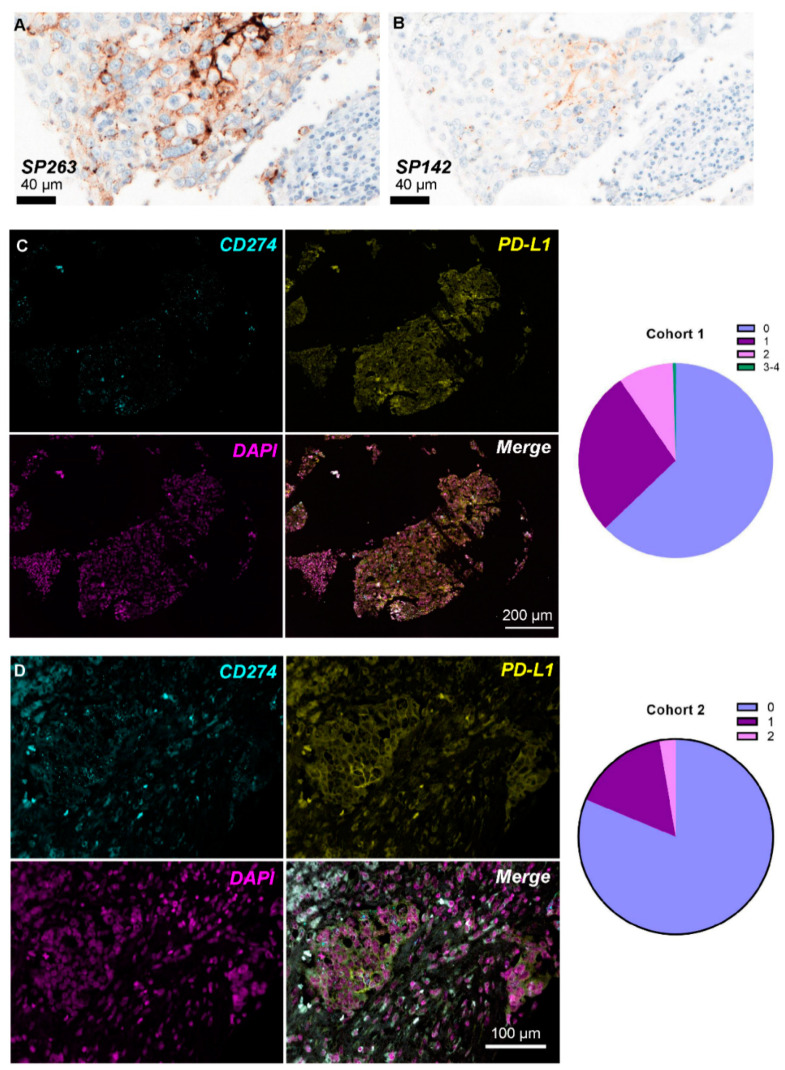
PD-L1 expression levels in breast cancer patient tissue. PD-L1 protein expression using IHC with the SP263 (**A**) and SP142 (**B**) Ventana antibody clones in the same patient in cohort 1. Representative images of concurrent PD-L1 mRNA-protein expression using combined RNA-FISH/IF method in the same FFPE patient sample in cohort 1 (**C**) and cohort 2 (**D**); the pie charts in the right panel represent the ACD RNAscope^®^ score distribution (0 = No staining or <1 dot/10 cells; 1 = 1–3 dots/cell; 2 = 4–9 dots/cell and none or very few dot clusters; 3 = 10–15 dots/cell and/or <10% dots are in clusters; 4 = >15 dots/cell and/or >10% dots are in clusters) per cohort.

Protein expression of PD-L1 was IHC evaluated in TMA cores of patients in cohort 1 with the commercially available antibody clones SP142 and SP263 (Figure 2A,B). Out of the 69 patients, 15/68 (22.0%) and 17/63 (27.0%) of those with available material were assessed as PD-L1-positive according to SP142 and SP263, respectively (71.4% concordance, Pearson chi-square *p* = 0.092). Patients with ER-negative BC were more often PD-L1-positive as assessed by SP263 (Pearson chi-square *p* = 0.001) and SP142 (Pearson chi-square *p* = 0.001). PD-L1 was positive in tumor and/or immune cells in 21/128 (16.4%) evaluable cores for SP142 and 23/118 (19.5%) for SP263 antibody. Protein expression according to the two antibody clones was concordant in 98/113 (86.7%) matched cores (Table 1) and significantly correlated (Pearson chi-square *p* < 0.001).

### 3.4. Positivity Rates of PD-L1 Protein and mRNA Expression in BC Tissue

#### 3.4.1. Positivity Rate of PD-L1 IF Expression

In cohort 1, two different ROIs from 109 evaluable TMA cores were analyzed for PD-L1 IF protein expression. A median of 55 cells (range, 37–96) were counted in each ROI; a median of 3 cells were found to be PD-L1-positive (range, 0–46) and two-thirds of ROIs contained at least one PD-L1-positive cell (144/218; 66%). In absolute counts, the mean number of positive cells per ROI was 7.10, with a mean positivity rate of 12.75% cells per area. PD-L1 IF expression was concordant with IHC SP142 and SP263 in 49.6% and 37.0% (Table 1) cores in cohort 1, respectively.

In cohort 2, a total of 112 ROIs from 56 TMA cores were included in the analysis. A median of 50 cells (range, 12–70) were counted per ROI, and a median of 0 cells (range, 0–14) expressed PD-L1 protein. PD-L1 expression assessment by immunofluorescence showed that 48/112 (42.9%) ROIs contained at least one PD-L1-positive cell. Moreover, the mean number of PD-L1-positive cells per ROI was 1.82, with a mean positivity of 3.99% cells per ROI examined.

#### 3.4.2. Positivity Rate of PD-L1 RNAscope^®^ Expression

The semi-quantitative ACD score was used for the quantification of PD-L1 mRNA positivity (dots/cell) per area. According to the results presented in Table 2, the majority of scored ROIs in cohort 1 demonstrated an ACD score of 0 (137/218; 62.8%), corresponding to lack of PD-L1 mRNA expression or low levels of <1 dot/10 cells (Figure 2C). This distribution was even more apparent in cohort 2 since 91/112 (81.3%) of the scored areas had no or very low staining (Figure 2D).

### 3.5. Concordance of PD-L1 Protein and mRNA Levels

*Concordance between PD-L1 IHC/PD-L1 IF and mRNA expression according to RNAscope^®^:* PD-L1 mRNA positivity was concordant with protein positivity according to SP142 IHC in 63 (59.4%) and according to SP263 clone in 53 (54.6%) cores in cohort 1 (Table 1). In addition, PD-L1 mRNA status was concordant with IF expression in 64 (58.7%) cores and significantly correlated (Pearson chi-square *p* = 0.002). Similar results were noted in cohort 2, with 71.2% of TMA cores exhibiting concordant PD-L1 status (Pearson chi-square *p* < 0.001).

*Concordance between PD-L1 IHC/PD-L1 IF and bulk PD-L1 gene expression:* In cohort 1, *CD274* transcripts were not statistically significantly associated with protein expression according to SP142 (Mann–Whitney *p* = 0.357), SP263 (*p* = 0.512) or IF (Spearman’s rho = 0.200, *p* = 0.164).

### 3.6. PD-L1 Protein and mRNA Heterogeneity

#### 3.6.1. Protein Heterogeneity

*Different cores of same patient:* In cohort 1, PD-L1 protein expression (positive vs. negative) was concordant between the duplicate cores of the same patient in 50/60 of cases using SP263 IHC (Pearson chi-square *p* < 0.001) and 49/60 of cases using SP142 IHC (Pearson chi-square *p* = 0.003). At the cellular level using IF (% positive cells per core), PD-L1 positivity was moderately correlated between the two cores (Spearman’s rho = 0.45, *p* < 0.001).

*Different areas of same core:* Heterogeneity of PD-L1 protein expression was also investigated between two selected areas of the same TMA core using IF. Neither in cohort 1 (Mann–Whitney *p* = 0.330) nor in cohort 2 (Mann–Whitney *p* = 0.411) was any significant difference observed between the distributions of PD-L1 positivity. The level of PD-L1 protein expression in one ROI of each core was strongly correlated with the other ROI of the same core in both cohorts (cohort 1: Spearman’s rho = 0.73, *p* < 0.001; cohort 2: Spearman’s rho = 0.53, *p* < 0.001).

#### 3.6.2. mRNA Heterogeneity

*Different cores of same patient:* In cohort 1, when PD-L1 mRNA expression was evaluated using the semi-quantitative ACD method, expression at two cores of the same patient was significantly associated (*p* < 0.001).

*Different areas of same core:* Using ACD score, the concordance of PD-L1 expression between two ROIs of the same core was 85.3% (93/109 concordant cores) in cohort 1, with the ACD values between the two ROIs being correlated (*p* < 0.001). Similar results were noted in cohort 2 (78.5% concordance, *p* < 0.001).

### 3.7. PD-L1 Expression in Different Cell Populations and Cellular Compartments

**Figure 3:**
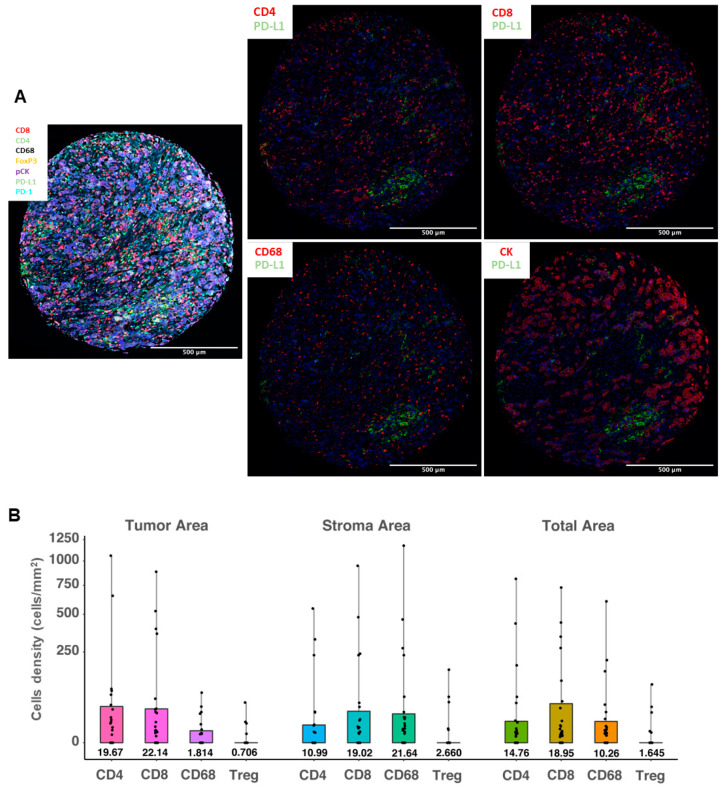
PD-L1 expression levels in different immune cell types using a multiplex fluorescent immunohistochemistry method. (**A**) Representative images of the multispectral imaging (overlay: left panel; co-expression: middle and right panels) using multiplex fluorescent IHC; (**B**) distribution of cell densities (cells/mm^2^, scale is square-root transformed for visualization purposes) of the PD-L1-positive immune cells in the tumor (left panel), stroma (middle panel) and total (right panel) areas, analyzed in 109 tissue samples. Boxes show median values and interquartile range, and whiskers demonstrate minimal and maximal values.

In order to further evaluate the cell origin of PD-L1 protein expression, we applied a multiplex fluorescent IHC method using different immune cell and epithelial markers. PD-L1 mean cell density in the total area (measured in cells/mm^2^) as assessed by multiplex fluorescent IHC was significantly correlated with PD-L1 IHC assessment by both SP142 (Mann–Whitney *p* = 0.008) and SP263 (*p* = 0.012). We also assessed the co-expression of PD-L1 and markers that define specific immune cell subpopulations: CD4 and CD8 for lymphocytes, FoxP3 for regulatory cells, and CD68 for macrophages, as well as PD-L1 expression in cytokeratin-positive tumor cells. Both the PD-L1 mean cell density in the total area and in different cellular compartments (stroma versus tumor) were assessed (Figure 3A). Overall, PD-L1 was mainly expressed in lymphocytes (mean cell density of PD-L1+/CD4+ cells: 14.7 cells/mm^2^ and of PD-L1+/CD8+ cells: 18.9 cells/mm^2^). However, in the stromal compartment, macrophages were the most common PD-L1-expressing cells (mean cell density of PD-L1+/CD68+ cells: 21.6 cells/mm^2^), followed by cytotoxic lymphocytes (mean cell density of PD-L1+/CD8+ cells: 19.0 cells/mm^2^). As expected, PD-L1-expressing tumor cells were dominant in tumor-rich areas, followed by infiltrating lymphocytes. The mean cell density of PD-L1+/CK+ cells was 153.1 cells/mm^2^, significantly higher than the density of PD-L1+/CD8+ cells (22.1 cells/mm^2^, Wilcoxon signed-rank *p* < 0.001) or PD-L1+/CD4+ cells (19.6 cells/mm^2^, *p* < 0.001). In addition, the density of PD-L1+/CD68+ cells in the tumoral compartment was significantly lower than in the stroma (*p* = 0.001). PD-L1-positive T regulatory cells (FoxP3+) were uncommon: mean cell density of 2.6 cells/mm^2^ in the stromal and 0.7 cells/mm^2^ in the tumoral compartment (Figure 3B).

## 4. Discussion

While immune checkpoint inhibition is routinely used in the therapeutic management of BC, the need for robust biomarkers remains unmet. Emerging data on PD-L1 protein expression denote its discrepant predictive value [7,20] and highlight the analytical difficulties of its assessment (different PD-L1 antibody clones, cut-offs, scoring methods), further indicating that PD-L1 positivity depends on assay performance [21] rather than true expression. In the present study, we evaluated the level of concordance and spatial heterogeneity of PD-L1 expression using a multi-level methods approach, demonstrating substantial discordance among the different methodologies. In comparison, previous comparative studies have been limited in that they mostly evaluated the PD-L1 IHC expression using different antibody clones with the sole focus on TNBC [22,23]. In line with previous results [6], we show here that PD-L1 positivity was highly concordant between SP142 and SP263 immunohistochemical antibodies, while the observed level of discordance varied depending on the applied method.

In our previous work, we have shown that PD-L1 gene expression is a positive prognostic factor for early BC, whereas protein expression in tumor cells is a negative one [10]. In this study, we investigated further this discrepancy and showed a non-significant correlation between protein and bulk mRNA expression, although in a small sample size. As bulk tumor analysis does not provide information about the specific cell types that express a gene of interest and does not account for the spatial heterogeneity of expression, we here performed RNA in situ hybridization analysis. We first demonstrated the analytical validity and feasibility of RNAscope^®^ for the assessment of PD-L1 mRNA expression in BC FFPE tissue, also in line with the current literature in breast [24,25,26,27] (Table S2) and other cancer types [28,29,30,31], as well as the combined detection of the protein, thus enabling the simultaneous visualization of gene and protein levels in situ. We observed that protein and mRNA expression was modestly correlated with each other, with the strength of the correlation being highly dependent on the evaluation method. Taken together, the aforementioned observations support our speculation that both analytical weaknesses and post-translational modifications can explain the seemingly discrepant results on the expression of PD-L1 and its prognostic value in BC.

Nevertheless, the question of which cells mainly express PD-L1 and carry the prognostic information remains, also considering the biological complexity of PD-1/PD-L1 regulation [32] and tumor-host interactions in BC [33]. In order to further elucidate the cell origin of PD-L1, we used a multiplex immunofluorescence assay for immune cell subpopulations and portrayed the expression patterns in different tissue compartments. Intratumoral PD-L1 was mostly expressed by lymphocytes (CD4+, CD8+ T-cells), while its stromal expression was observed mostly in macrophages. Due to the small size of the study population, any potential clinical implications need to be tested in larger cohorts. However, the significant correlation of multiplex fluorescent IHC with the IHC assays observed in this and other studies [34,35], and the increased spatial capacity and improved performance over other biomarker modalities [36], underscore the strong potential of multiplexed methodologies for a deeper understanding of the immune infiltrate.

Previous studies support that inter-assay discordance of PD-L1 expression is likely attributable to other factors than antibody epitope [37], pointing toward assay/platform performance as well as intratumoral heterogeneity (ITH). Regarding the latter, heterogeneity both at the genetic and epigenetic level has been proposed to influence many aspects of tumor biology [38] and clinical behavior, including resistance to pharmacologic therapies [39], thus becoming the driving force that promotes clonal expansion and phenotypic diversification [40]. This is of paramount importance since increased heterogeneity in the expression of a therapeutic target such as HER2 [41] and PD-L1 could signify a lower response rate to targeted therapies. Previous reports on PD-L1 heterogeneity have focused on the intra-patient temporal expression of PD-L1 protein between primary tumors and metastatic sites, demonstrating a differential expression pattern and correlation with outcome [9,42,43,44]. Of note, a recent study reported substantial heterogeneity of PD-L1 expression between different areas of the same tissue in TNBC patients, especially for small tumor samples [45]. Similar results have been demonstrated in other cancer types [46]. Based on our results, we could not conclusively demonstrate the heterogeneity of PD-L1 expression neither at protein nor mRNA level. However, the moderate concordance and correlation between two ROIs of the same TMA core and the absence of total concordance between cores provide a possible hint toward spatial heterogeneity.

This largely exploratory study suffers from several limitations. First, the number of samples included in the analysis was relatively small, which may have masked potential associations or led to associations by chance. Moreover, the possible presence of false positives or negative samples, especially for the RNA signal, could not be excluded since the evaluation of RNA was performed in a semi-quantitative and not in an automated way, and no optimal cut-off or method has been established for both fluorescent protein and RNA-FISH signal quantification. Another limitation of the used combined mRNA-protein method is that the origin of PD-L1 expression could not be distinguished between tumor and immune cell, a limitation circumvented by the use of the multiplex methodology. Furthermore, the evaluation of PD-L1 at the protein level was performed in TMA. Previously published studies have shown that assessment of the immune infiltrate in TMA underestimates its extent [47,48], which may have been one main cause why PD-L1 heterogeneity was not demonstrated in our experiments. Thus, due to the aforementioned inherent weaknesses of the study, cautious interpretation of our results is warranted.

## 5. Conclusions

In conclusion, we evaluated the level of heterogeneity of PD-L1 expression in early BC through a comprehensive multi-level analysis. To our knowledge, this is the first study evaluating the degree of concordance of PD-L1 spatial expression both at the protein and gene expression levels, using multiple methodologies. We demonstrated a discordance of expression among the used methods and between different areas of the same tumor, which hints toward the presence of spatial, intratumoral, and biological heterogeneity. Improving the evaluation methods and delineating potential PD-L1 heterogeneity in the tumor-immune microenvironment may provide crucial insights into the underlying biology that governs treatment response. These hypothesis-generating results, if independently validated, may have significant clinical implications: PD-L1 protein expression, the only predictive biomarker currently in clinical use, may not be the optimal tool for patient selection for immunotherapy. Combined mRNA-protein or multiplex methodologies may portray the underlying tumor-host interactions more accurately and predict benefit from checkpoint inhibition, albeit prospective validation is needed.

## Figures and Tables

**Table 1:** PD-L1 protein and mRNA expression positivity rates and concordance in cohort 1 at core level using a common cut-off for all methodologies. Cases were classified as PD-L1-positive if at least one cell was stained for PD-L1. +/+ indicates positivity for the marker at the vertical and horizontal axis, while −/− indicates lack of expression. When the same marker is on both axes, it indicates the total number of positives (+/+) and negatives (−/−) according to the specific marker, and no concordance is provided. Cases that stained positive with one marker and negative with another are not depicted.

Methodology	SP142 *n* (%)				SP263 *n* (%)				IF *n* (%)				RNAscope *n* (%)				MFI *n* (%)			
	+/+	−/−	Concordant	*p*-Value *	+/+	−/−	Concordant	*p*-Value *	+/+	−/−	Concordant	*p*-Value *	+/+	−/−	Concordant	*p*-Value *	+/+	−/−	Concordant	*p*-Value *
SP142	21 (16.4)	107 (83.6)	-	-	14 (12.3)	84 (74.3)	108 (86.6)	<0.001	18 (17.0)	24 (22.6)	42 (49.6)	0.038	10 (9.4)	53 (50.0)	63 (59.4)	0.277	12 (11.4)	53 (50.5)	65 (61.9)	0.032
SP263	14 (12.3)	84 (74.3)	108 (86.6)	<0.001	23 (19.5)	95 (70.9)	-	-	18 (18.5)	18 (18.5)	36 (37)	0.355	10 (10.3)	43 (44.3)	53 (54.6)	0.732	12 (12.2)	49 (50.0)	61 (62.2)	0.046
IF	18 (17.0)	24 (22.6)	42 (49.6)	0.038	18 (18.5)	18 (18.5)	36 (37)	0.355	83 (76.1)	26 (23.9)	-	-	42 (38.5)	22 (20.2)	64 (58.7)	0.002	35 (35.3)	15 (15.1)	50 (50.5)	0.432
RNAscope	10 (9.4)	53 (50.0)	63 (59.4)	0.277	10 (10.3)	43 (44.3)	53 (54.6)	0.732	42 (38.5)	22 (20.2)	64 (58.7)	0.002	46 (42.2)	63 (57.8)	-	-	14 (14.1)	27 (27.2)	41 (41.4)	0.056
MFI	12 (11.4)	53 (50.5)	65 (61.9)	0.032	12 (12.2)	49 (50.0)	61 (62.2)	0.046	35 (35.3)	15 (15.1)	50 (50.5)	0.432	14 (14.1)	27 (27.2)	41 (41.4)	0.056	48 (44.4)	60 (55.5)	-	-

* Pearson chi-square. Abbreviations: IF: immunofluorescence; MFI: multiplex fluorescent immunohistochemistry.

**Table 2:** PD-L1 mRNA positivity rates per area examined in the two clinical cohorts according to a semi-quantitative method (dots/cell).

ACD Score	Cohort 1: Number of Areas Scored (%)	Cohort 2: Number of Areas Scored (%)
0	137 (62.8%)	91 (81.3%)
1	60 (27.5%)	18 (16.1%)
2	20 (9.2%)	3 (2.7%)
3	1 (0.5%)	0
4	0	0
TOTAL	218 (100%)	112 (100%)

## Data Availability

Gene expression data are available at the Gene Expression Omnibus (GEO) database under accession number GSE48091. Further data that support the findings of this study are available from the corresponding author upon reasonable request.

## Supplementary Materials

The following are available online at https://www.mdpi.com/article/10.3390/cancers13184655/s1, Table S1: Patient baseline characteristics of included patients from the 2 clinical cohorts; Table S2: Overview of the studies evaluating PD-L1 expression using the RNAscope^®^ technology in breast cancer; Figure S1: RNAscope assay using 3 positive control probes (POLR2A, PPIB, UBC) in HeLa human cells. Original magnification 630× Figure S2: Experimental workflow of RNAscope^®^ combined with immunofluorescence (IF) for the simultaneous detection of PD-L1 mRNA and protein expression, respectively in the same BC FFPE tissue section; Figure S3: Overview and flowchart of the different methods applied in the two study cohorts (adjuvant and neoadjuvant).
