# Supplementary material for: Discordance of PD-L1 Expression at the Protein and RNA Levels in Early Breast Cancer
Source: Cancers (Basel). 2021 Sep 16;13(18):4655. doi: 10.3390/cancers13184655 (PMC8467035; doi:10.3390/cancers13184655)
Supplement: Supplementary file 1 [file cancers-13-04655-s001.zip › cancers-1324496-supplementary.pdf]

cancers

MDPI

Supplementary Material: Discordance of PD-L1 Expression at the Protein and RNA Levels in Early Breast Cancer

Ioannis Zerdes, Vaia Karafousia, Artur Mezheyeuski, Maria Stogiannitsi, Raoul Kuiper, Pablo Moreno Ruiz, George Rassidakis, Jonas Bergh, Thomas Hatschek, Theodoros Foukakis and Alexios Matikas

Table S1. Patient baseline characteristics of included patients from the 2 clinical cohorts.

Characteristic	Cohort 1 (Merck, *n* = 69)	Cohort 2 (PROMIX, *n* = 36)
**Grade**		
G1	5	2
G2	22	11
G3	28	10
Unknown	14	13
**ER status**		
Positive	38	21
Negative	17	8
Unknown	14	7
**PR status**		
Positive	30	20
Negative	18	12
Unknown	21	4
**HER2 status**		
Positive	7	0
Negative	35	25
Unknown	27	11
**Pathologic response**		
pCR	NA	4
no pCR	NA	32
**Age**		
Median (range)	55.5 (28.0–74.9)	49.7 (33.7–69.2)
**Tumor size in mm**		
Median (range)	22 (10–55)	60 (30–180)
**Lymph node status**		
Positive	27	22
Negative	28	4
Unknown	14	10

Abbreviations: ER: estrogen receptor; PR: progesterone receptor; HER2: human epidermal growth factor receptor; pCR: pathologic complete response; NA: not available.

*The lymph node status/size for patients in cohort 2 has been estimated at the time of diagnosis (baseline).

Table S2. Overview of the studies evaluating PD-L1 expression using the RNAscope® technology in breast cancer.

First author	Year	Journal	Number of patients (subtype)	RNAscope® assay and scoring method	RNAscope® expression	IHC/IF Ab clone	Comments
Schalper K. et al.	2013	Clinical Cancer Research	636	Manual chromogenic RNAscope® assay coupled to automated quantitative fluorescence (QIF) detection / AQUA® method	≈ 60% (Any expression >noise threshold of a DapB negative control quantification)	clone 5h1 (QIF detection / AQUA®)	▪ PD-L1 mRNA expression was significantly correlated with longer recurrence-free survival<br>▪ PD-L1 mRNA was weakly significantly correlated with protein expression in the 2 TMA sets (R=0.20, p=0.01; R=0.16, p=0.003)
Guo L. et al.	2016	SpringerPlus	183 (TNBC)	RNAscope® 2.0 High Definition-BROWN assay	NA	SP142 (Ventana)	▪ High concordance between PD-L1 protein and mRNA expression
Humphries M. et al.	2018	Journal of Oncology	109 (ER-/HER2+)	Manual chromogenic RNAScope® assay / Spotstudio Software from ACD	NA	SP142 (Ventana)	▪ Non-significant trend of high PD-L1 mRNA expression (cut-off: median) towards improved relapse-free and overall survival
Ren X. et al.	2018	Cancer Biology & Therapy	101 (TNBC)	RNAscope® FFPE 2.0 HD detection kit (Brown) / ACD scoring system (dots/cell; scale 0-4);	74.4% in tumor cells and 50.9% in TILs (Positivity if ACD score>0)	SP263 (Ventana)	▪ Kappa value for PD-L1 in tumor cells protein: 0.061 and PD-L1 in TILs: 0.234<br>▪ PDL1 mRNA expression in tumor cells was significantly related PDL1 mRNA positivity in TILs (p=0.006)<br>▪ No significant correlation to recurrence or survival

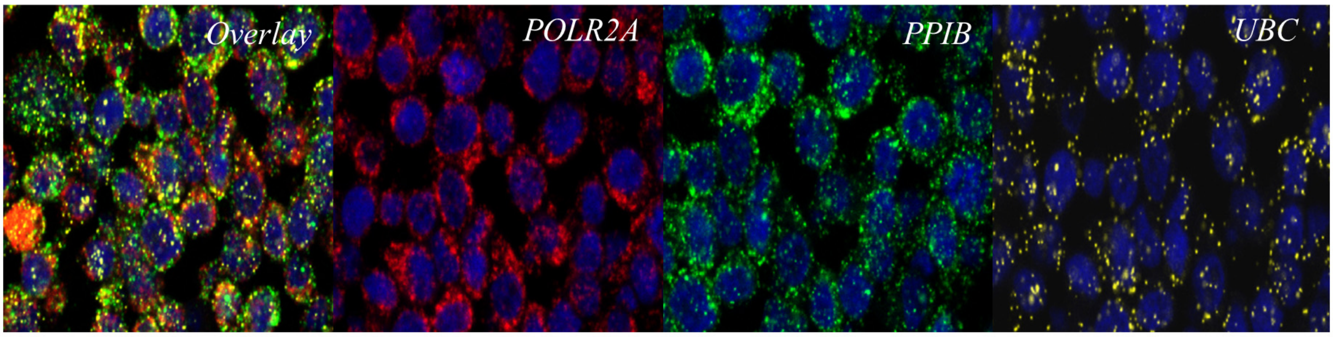

Figure S1. RNAscope assay using 3 positive control probes (POLR2A, PPIB, UBC) in HeLa human cells. Original magnification x 630.

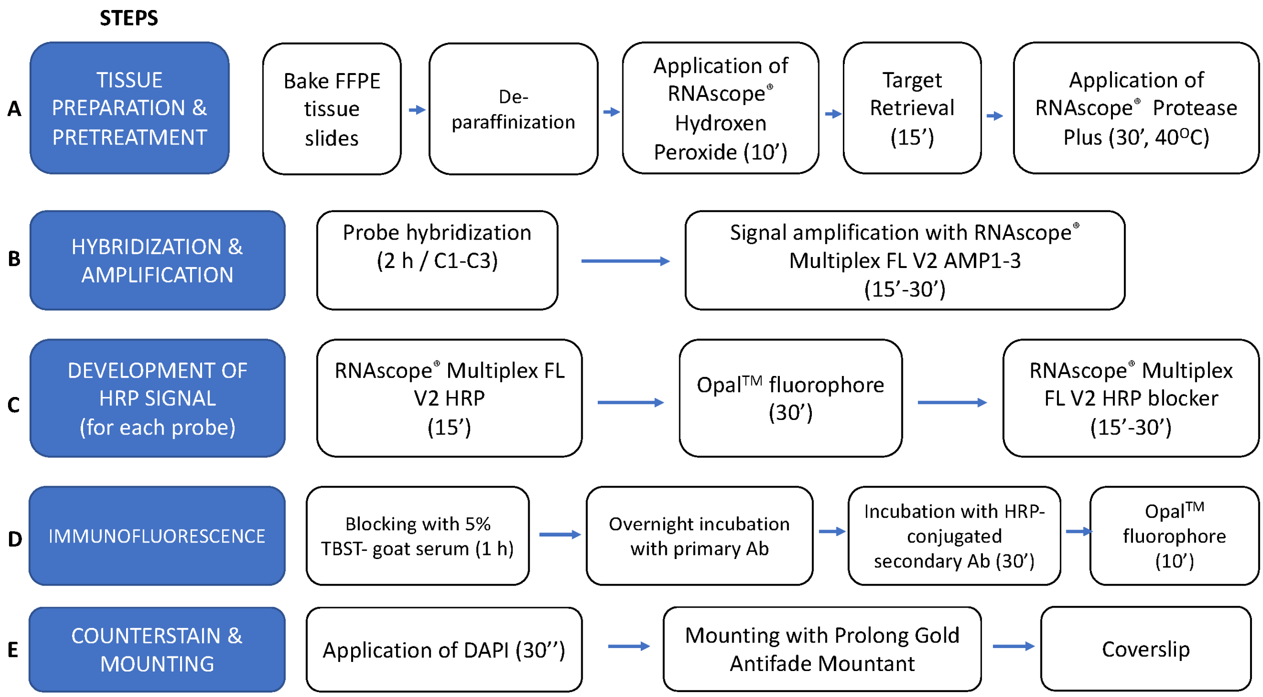

Figure S2. Experimental workflow of RNAscope® combined with immunofluorescence (IF) for the simultaneous detection of PD-L1 mRNA and protein expression, respectively in the same BC FFPE tissue section.

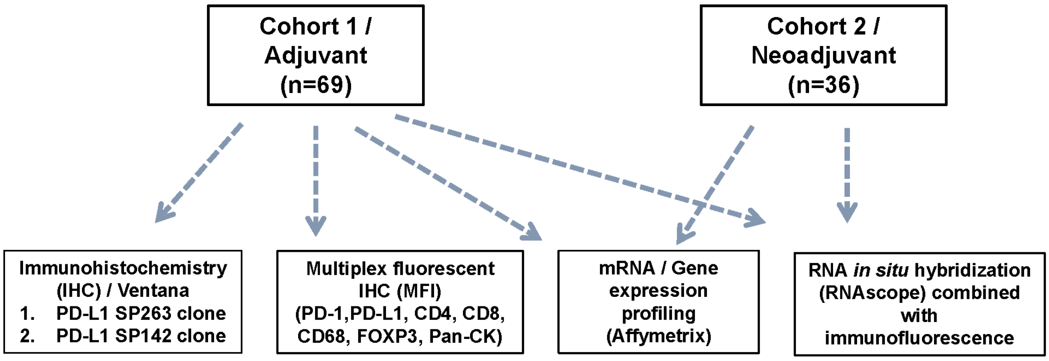

Figure S3. Overview and flowchart of the different methods applied in the two study cohorts (adjuvant Cohort 1; neoadjuvant Cohort 2).
